# Major adverse cardiovascular events of enzalutamide versus abiraterone in prostate cancer: a retrospective cohort study

**DOI:** 10.1038/s41391-023-00757-0

**Published:** 2023-12-05

**Authors:** Yan Hiu Athena Lee, Jeremy Man Ho Hui, Chi Ho Leung, Christopher Tze Wei Tsang, Kyle Hui, Pias Tang, Jeffrey Shi Kai Chan, Edward Christopher Dee, Kenrick Ng, Sean McBride, Paul L. Nguyen, Gary Tse, Chi Fai Ng

**Affiliations:** 1 Cardio-Oncology Research Unit, Cardiovascular Analytics Group, PowerHealth Research Institute, Hong Kong, China; 2 Division of Urology, Department of Surgery, Faculty of Medicine, The Chinese University of Hong Kong, Hong Kong, China; 3 Department of Radiation Oncology, Memorial Sloan Kettering Cancer Center, New York, NY USA; 4 Department of Medical Oncology, University College London Hospitals NHS Foundation Trust, London, UK; 5 Department of Radiation Oncology, Dana-Farber/Brigham and Women’s Cancer Center and Harvard Medical School, Boston, MA USA; 6 Tianjin Key Laboratory of Ionic-Molecular Function of Cardiovascular Disease, Department of Cardiology, Tianjin Institute of Cardiology, Second Hospital of Tianjin Medical University, Tianjin, 300211 China; 7 Kent and Medway Medical School, Canterbury, Kent CT2 7NT UK; 8 School of Nursing and Health Studies, Hong Kong Metropolitan University, Hong Kong, China; 9 SH Ho Urology Centre, The Chinese University of Hong Kong, Hong Kong, China

**Keywords:** Urological cancer, Urological cancer, Prostate cancer

## Abstract

**Background:**

While the cardiovascular risks of androgen receptor pathway inhibitors have been studied, they were seldom compared directly. This study compares the risks of major adverse cardiovascular events (MACE) between enzalutamide and abiraterone among prostate cancer (PCa) patients.

**Methods:**

Adult PCa patients receiving either enzalutamide or abiraterone in addition to androgen deprivation therapy in Hong Kong between 1 December 1999 and 31 March 2021 were identified in this retrospective cohort study. Patients who switched between enzalutamide and abiraterone, initiated abiraterone used without steroids, or experienced prior cardiac events were excluded. Patients were followed-up until 30 September 2021. The primary outcomes were MACE, a composite of stroke, myocardial infarction (MI), Heart failure (HF), or all-cause mortality and a composite of adverse cardiovascular events (CACE) not including all-cause mortality. The secondary outcomes were individual components of MACE. Inverse probability treatment weighting was used to balance covariates between treatment groups.

**Results:**

In total, 1015 patients were analyzed (456 enzalutamide users and 559 abiraterone users; mean age 70.6 ± 8.8 years old) over a median follow-up duration of 11.3 (IQR: 5.3–21.3) months. Enzalutamide users had significantly lower risks of 4P-MACE (weighted hazard ratio (wHR) 0.71 [95% confidence interval (CI) 0.59–0.86], *p* < 0.001) and CACE (wHR 0.63 [95% CI: 0.42–0.96], *p* = 0.031), which remained consistent in multivariable analysis. Such an association may be stronger in patients aged ≥65 years or without diabetes mellitus and was independent of bilateral orchidectomy. Enzalutamide users also had significantly lower risks of MI (wHR 0.57 [95% CI: 0.33–0.97], *p* = 0.040) and all-cause mortality (wHR 0.71 [95% CI: 0.59–0.85], *p* < 0.001).

**Conclusion:**

Enzalutamide was associated with lower cardiovascular risks than abiraterone in PCa patients.

## Introduction

Globally, prostate cancer (PCa) is the second most common cancer and fifth major cause of cancer mortality among males in 2020 [1]. Androgen deprivation therapy (ADT) has been a standard treatment for PCa. However, PCa may progress to castration-resistant PCa (CRPC) within several years of ADT initiation because of tumor escape mechanisms [2]. Currently, androgen receptor pathway inhibitors (ARPI) such as enzalutamide and abiraterone acetate are used as one of the first-line treatments for metastatic CRPC. Enzalutamide and abiraterone are well-tolerated and improve survival when used with ADT. Furthermore, they improve treatment efficacy when used in the early stages of PCa [3–5].

Nonetheless, with various ARPIs available, it is important to consider their risk profiles in treatment decision-making. While greater treatment response rates and survival benefits were observed among enzalutamide users when compared to abiraterone users [6, 7], their risks of adverse cardiac events were seldom compared. In a recent meta-analysis of prospective studies, abiraterone was found to have significantly higher cardiotoxicity than enzalutamide [8]. Nonetheless, observational studies which might provide further insight into the cardiac risks of the two drugs in real-world practice are lacking. Furthermore, most studies do not provide the comparative risk of individual adverse cardiac events such as stroke, myocardial infarction (MI) and heart failure (HF). Therefore, the aim of this study was to compare the risks for major adverse cardiac events (MACE) between enzalutamide and abiraterone use among patients with PCa.

## Methods

### Source of data

This retrospective cohort study has been approved by the Joint Chinese University of Hong Kong—New Territories East Cluster Clinical Research Ethics Committee. It was performed in accordance with the STROBE guideline.

All data were retrieved from the Clinical Data Analysis and Reporting System (CDARS), a population-based electronic health records database documenting key demographics, diagnoses, procedures, and medication records of patients attending public healthcare institutions in Hong Kong. All diagnoses are coded by the *International Classification of Diseases, Ninth Revision* (ICD-9) codes. CDARS is linked to the Hong Kong Death Registry, a governmental registry of all Hong Kong citizens’ death records, from which mortality data may be obtained. Causes of mortality were encoded using either ICD-9 or ICD-10 codes, depending on the year of death. This system has been used extensively for conducting epidemiological studies by local teams [9, 10]. Our team has previously studied the cardiovascular burden and the effects of medications in prostate cancer patients who received ADT [11, 12].

### Study design and population

Adult patients (18 years old or above) diagnosed with PCa who were using enzalutamide or abiraterone in addition to ADT in Hong Kong between 1 December 1999 and 31 March 2021 were included. Diagnosis of PCa was determined by ICD-9 codes (Supplementary Table 1). ADT included bilateral orchidectomy (BO), gonadotrophin-releasing hormone agonists, and gonadotrophin-releasing hormone antagonists.

The following patients were excluded: (a) with both drugs in their treatment course, (b) with the initiation of abiraterone use without steroids, (c) with prior stroke, MI, and heart failure HF.

### Follow-up and outcomes

All patients were followed-up from the day of enzalutamide or abiraterone initiation (baseline date) until 30 September 2021. The primary outcome were new-onset 4-point MACE (4P-MACE) defined as the first occurrence of stroke, MI, HF, or all-cause mortality; it is one of the most common MACE definitions used in observational studies [13], as well as a composite of adverse cardiovascular events (CACE) defined as the first occurrence of stroke, MI or HF to further demonstrate the cardiovascular morbidities associated with enzalutamide/abiraterone. The secondary outcomes were the individual components of 4P-MACE. Patients without 4P-MACE events were censored at last follow-up. All causes of death were determined by ICD codes (Supplementary Table 1).

### Statistical analyses

All patients’ age and other comorbidities at baseline (hypertension, diabetes, hyperlipidaemia, ischemic heart disease, chronic kidney disease, and other malignancies), and the use of other medications (anticoagulant and steroid), use of other treatments of PCa (prostatectomy, radiotherapy, chemotherapy), duration of prior ADT, and prostate-specific antigen (PSA) levels at baseline were recorded. The corresponding ICD-9 diagnostic codes were shown in Supplementary Table 1.

Continuous variables were expressed as mean ± standard deviation, while categorical variables were presented as number (percentage). To account for the confounding effects of the aforementioned covariates, we calculated propensity scores (PS) and applied inverse probability of treatment weighting (IPTW) to estimate the average treatment effect on the treated (ATT). PSA level at baseline was log-transformed before PS calculation. Generalized boosted models (GBM) were used to estimate the propensity scores, and we minimized the largest absolute standardized mean differences (ASMD) of the covariates to choose the optimal iteration [14]. ASMD were calculated for each covariate to examine the balance of covariates between treatment groups, with values < 0.1 being considered to represent good balance. Multivariable analysis was also performed with the same set of covariates.

Cox regression was used to estimate the weighted hazard ratios (wHR) of 4P-MACE and the 95% confidence intervals (CI). For multivariable analysis, the summary statistics were adjusted hazard ratios (aHR) and 95% CI. Schoenfeld test did not show any violation of the proportional hazards assumption. Sensitivity analysis based on PS trimming at 1%, 2.5%, and 5% was performed to assess bias from unmeasured confounding [15]. Kaplan–Meier curves were used to estimate 4P-MACE-free survival in the weighted cohort.

All *p* values were two-sided, with values less than 0.05 considered statistically significant. All statistical analyses were performed using R software, version 4.2.0 (R Foundation for Statistical Computing).

### Subgroup analyses

An a priori subgroup analysis was performed, comparing patients aged <65 and ≥65 years, to explore whether the differences in cardiac risks between enzalutamide and abiraterone are affected by age. A second a priori subgroup analysis was performed for patients with or without baseline diabetes mellitus to investigate whether the observed differences are mediated by glycaemic control. A third a priori subgroup analysis was performed for patients with or without BO, as BO may be associated with adverse cardiovascular events [16].

### Sensitivity analyses

Sensitivity analysis using PS trimming was performed to demonstrate the robustness of our findings.

## Results

### Study cohort

**Fig. 1:**
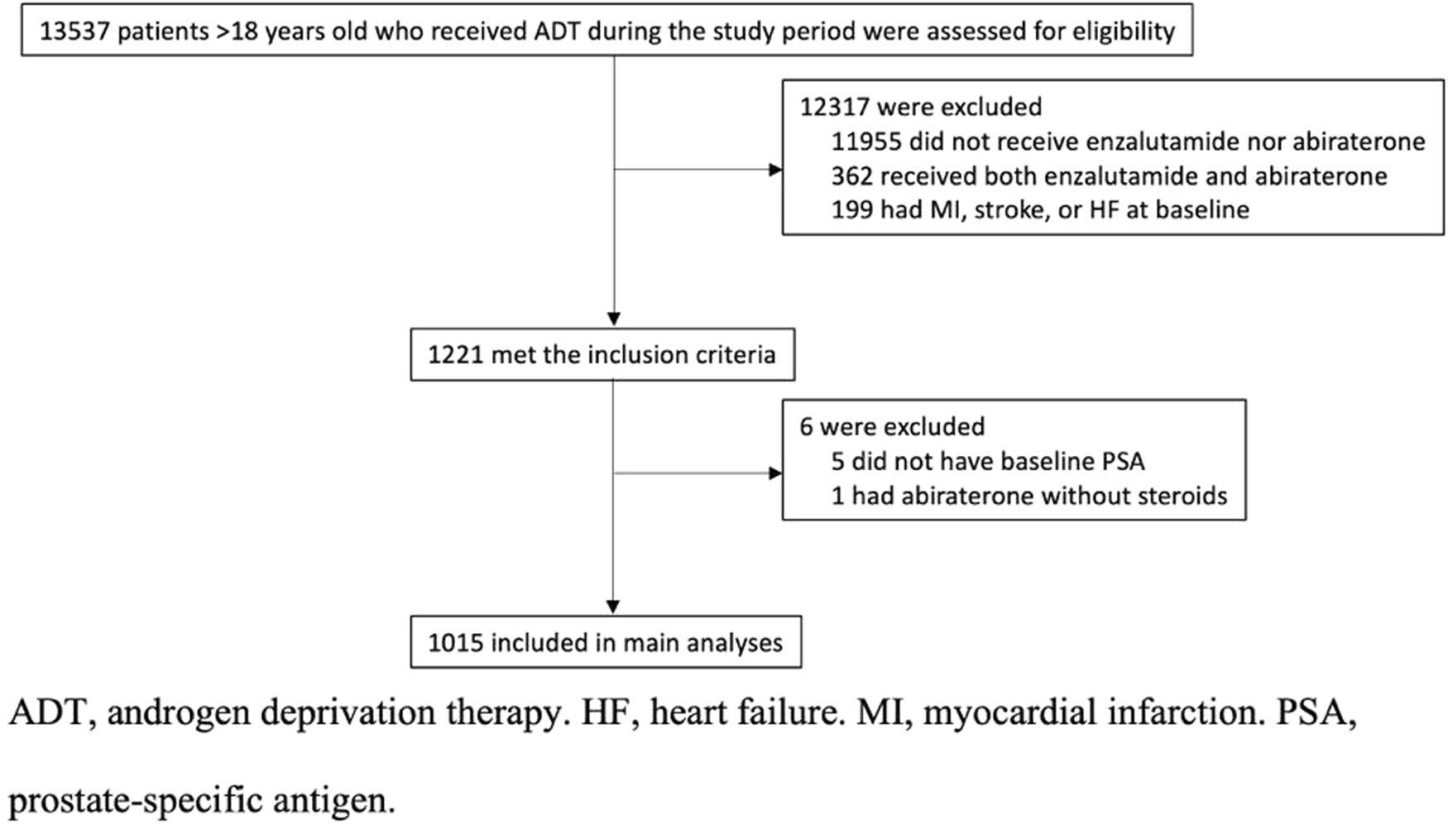
Study flow chart for patient identification, inclusion and exclusion.

In total, 13,537 adult patients with PCa who received ADT were identified. After excluding patients without enzalutamide or abiraterone use (*n* = 11,955), those with both enzalutamide or abiraterone use (*n* = 362), with the initiation of abiraterone use without steroids (*n* = 1), with prior stroke, MI, or HF (*n* = 199), or without baseline PSA (*n* = 5), 1015 patients (mean age 70.6 ± 8.8 years old) were included in the final analysis (Fig. 1), of whom 456 were enzalutamide users over a median duration of treatment of 6.5 (IQR 3.0–12.3) and 559 were abiraterone users over a median duration of treatment of 5.5 (IQR 1.9–12.2). Baseline characteristics of included patients were summarized in Table 1, which also demonstrates good balance of all covariates by IPTW (ASMD < 0.1 for all).

**Table 1:** Baseline characteristics and balancing diagnostics before and after IPTW.

Characteristics	All (*N* = 1015)	Abiraterone (*n* = 559)	Enzalutamide (*n* = 456)	ASMD before IPTW	*P*-value before IPTW	ASMD after IPTW	*P*-value after IPTW
Age, years	70.6 ± 8.8	69.7 ± 8.7	71.7 ± 8.8	0.23	<0.001	0.01	0.899
Diabetes, *n* (%)	306 (30.1)	130 (23.3)	176 (38.6)	0.32	<0.001	0.07	0.055
Hypertension, *n* (%)	804 (79.2)	430 (76.9)	374 (82.0)	0.13	0.047	0.02	0.571
Ischemic heart disease, *n* (%)	71 (7.0)	38 (6.8)	33 (7.2)	0.02	0.785	0.02	0.290
Chronic kidney disease, *n* (%)	31 (3.1)	17 (3.0)	14 (3.1)	0.002	0.979	0.006	0.588
Hyperlipidemia, *n* (%)	414 (40.8)	192 (34.3)	222 (48.7)	0.29	<0.001	0.06	0.078
Other malignancies, *n* (%)	55 (5.4)	32 (5.7)	23 (5.0)	0.03	0.634	0.02	0.365
Ever underwent prostatectomy, *n* (%)	138 (13.6)	70 (12.5)	68 (14.9)	0.07	0.269	0.008	0.762
Ever underwent radiotherapy, *n* (%)	21 (2.1)	13 (2.3)	8 (1.8)	0.04	0.525	0.004	0.704
Ever underwent chemotherapy, *n* (%)	384 (37.8)	221 (39.5)	163 (35.7)	0.08	0.216	0.01	0.677
Androgen deprivation therapy duration, months	38.9 ± 35.7	37.4 ± 36.5	40.6 ± 34.6	0.09	0.006	0.02	0.751
Anticoagulant use, *n* (%)	399 (39.3)	220 (39.4)	179 (39.3)	0.002	0.974	0.01	0.772
Prior steroid use, *n* (%)	562 (55.4)	356 (63.7)	206 (45.2)	0.37	<0.001	0.05	0.137
Prostate specific antigen*, ng/mL	366 ± 966	356 ± 732	379 ± 1192	0.13	0.014	0.06	0.362

*ASMD* absolute standardized mean difference, *IPTW* inverse probability of treatment weighting.

*ASMD was calculated based on log-transformed values.

### Outcomes

**Fig. 2:**
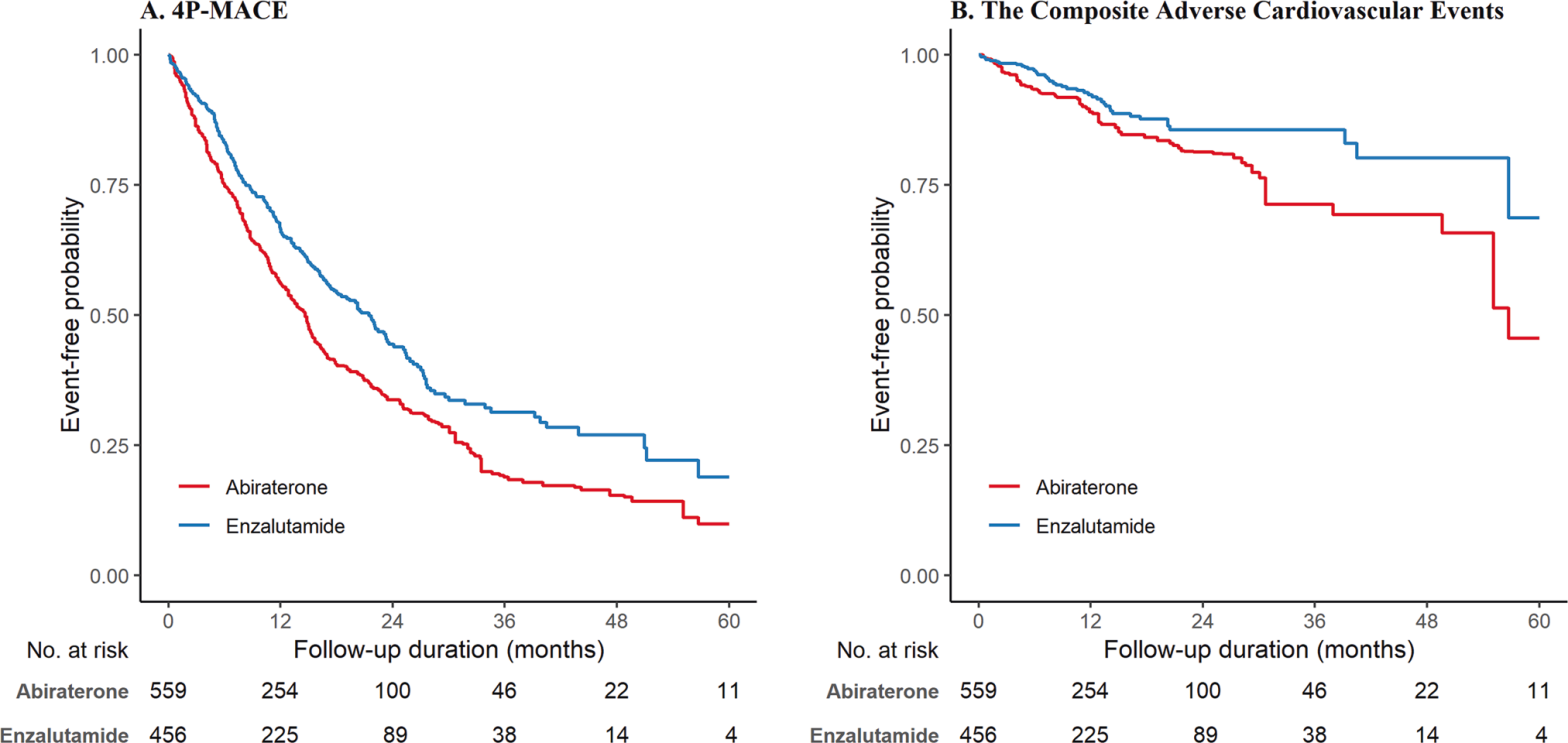
Weighted Kaplan–Meier survival curves. **A** 4P-MACE. **B** CACE. 4P-MACE: 4-point major adverse cardiovascular events. CACE composite adverse cardiovascular events.

**Fig. 3:**
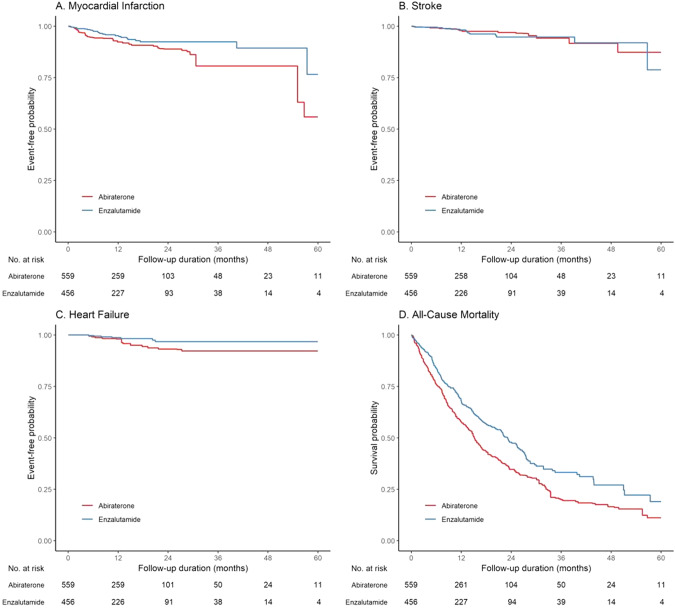
Weighted Kaplan–Meier survival curves for the individual components of 4P-MACE. **A** Myocardial infarction. **B** Stroke. **C** Heart failure. **D** All-cause mortality.

Over a median follow-up period of 11.3 (IQR: 5.3–21.3) months, 591 (58.2%) patients had 4P-MACE, 114 (11.2%) patients had the composite adverse cardiovascular event, 65 (6.40%) had MI, 37 (3.65%) had stroke, 21 (2.07%) had HF, 13 (1.3%) patients had CV mortality, 334 (32.9%) patients had cancer-related mortality, and 575 (56.7%) had all-cause mortality. The results of Cox regression are shown in Supplementary Table 2. Overall, enzalutamide users had significantly lower risks of 4P-MACE (wHR 0.71 [95% CI: 0.59–0.86], *p* < 0.001) and CACE (wHR 0.63 [95% CI: 0.42–0.96], *p* = 0.031) than abiraterone users, as visualized in the Kaplan–Meier curve in Fig. 2. This association remained consistent in the multivariable analysis (aHR 0.71 [95% CI: 0.60–0.84], *p* < 0.001) as shown in Supplementary Table 3. Enzalutamide users had significantly lower risks of MI (wHR 0.57 [95% CI: 0.33–0.97], *p* = 0.040) and all-cause mortality (wHR 0.71 [95% CI: 0.59–0.85], *p* < 0.001) than abiraterone users, as visualized in the Kaplan–Meier curves in Fig. 3.

### Subgroup analyses

**Fig. 4:**
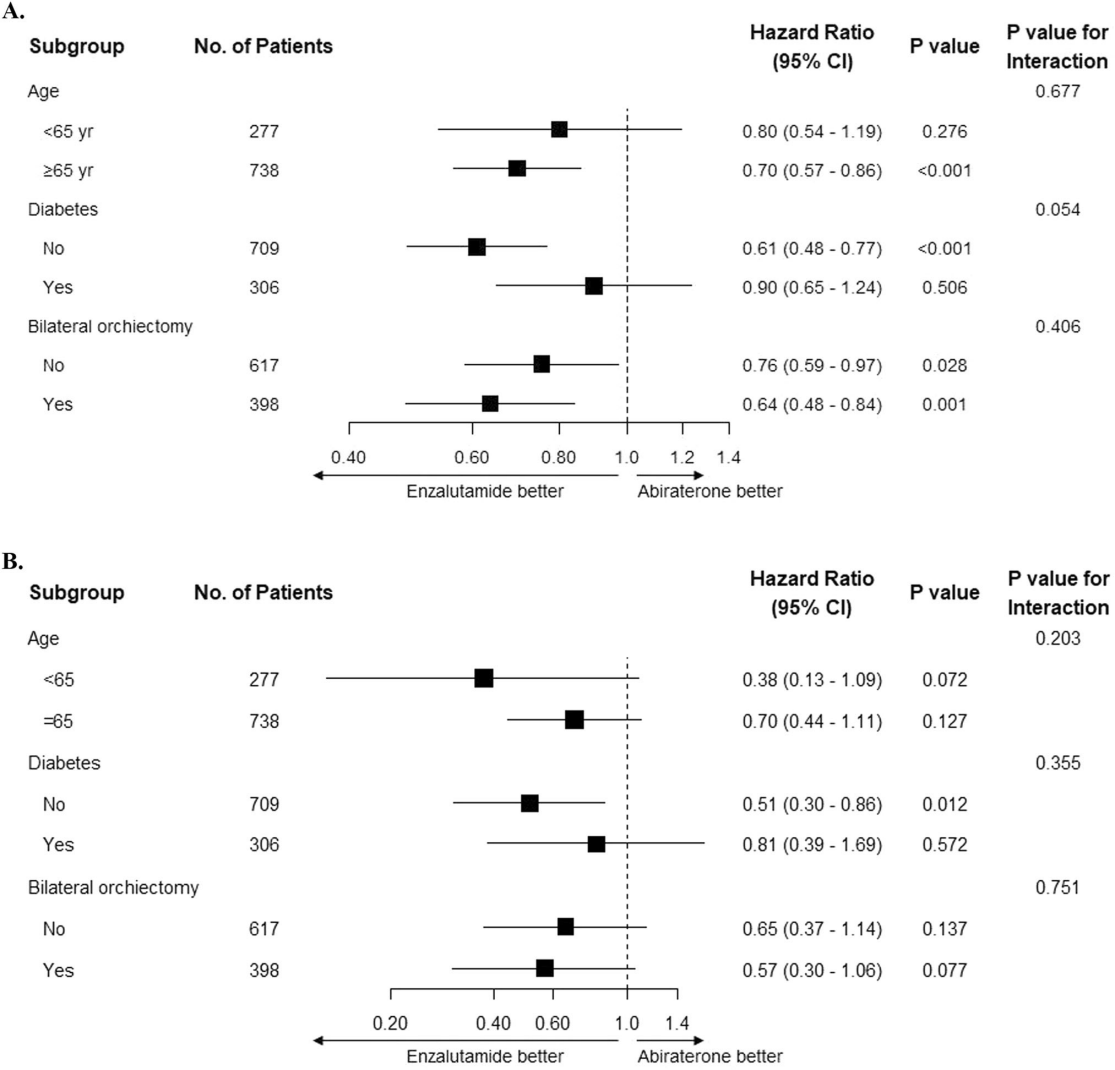
Subgroup analyses. **A** 4P-MACE. **B** CACE. Hazard ratios referenced against abiraterone use.

In the subgroup analysis by age of patients aged ≥65 years old (*n* = 738) or <65 years old (*n* = 277), only enzalutamide users aged ≥65 years old had significantly lower risks of 4P-MACE (*p*-value for interaction = 0.677). Among patients with or without DM (*n* = 306 and *n* = 709, respectively), only enzalutamide users without DM had significantly lower risks of 4P-MACE (*p*-value for interaction = 0.054). While the impact of DM could lead to similar cardiovascular risks between the two treatment groups, the subgroup-specific effects and borderline significant interaction effect may suggest that DM affects the observed associations between enzalutamide use and risks of 4P-MACE. Among both patients with or without BO (*n* = 398 and *n* = 617, respectively), enzalutamide users had significantly lower risks of 4P-MACE (*p*-value for interaction = 0.406). This suggested that BO did not affect the observed associations between enzalutamide use and risks of 4P-MACE. The results of subgroup analyses are summarized in Fig. 4.

### Sensitivity analyses

PS trimming showed that enzalutamide use was significantly associated with lower risks of 4P-MACE when the upper and lower 1%, 2.5%, and 5% of PS were trimmed (*p* < 0.01 for all). For the CACE, the result remained significant when the upper and lower 1% of PS were trimmed (wHR 0.62 [95% CI 0.40–0.98], *p* = 0.039).

## Discussion

This retrospective cohort study showed that, enzalutamide use was associated with significantly lower risks of 4P-MACE than abiraterone use.

### Potential mechanisms

Abiraterone selectively and irreversibly inhibits 17 alpha-hydroxylase/C17,20-lyase (CYP17A1), thereby suppressing androgen synthesis. However, the inhibition of CYP17A1 may also decreases cortisol level. The resulting negative feedback increases adrenocorticotropic hormone secretion and thus the synthesis of aldosterone. Increased aldosterone may lead to hypokalaemia, which predisposes to arrhythmia and HF [17]. To reduce the mineralocorticoid toxicity, current treatment guidelines recommend abiraterone to always be co-prescribed with steroids. Although co-prescription significantly lowers the cardiovascular risks, steroids may not fully counteract the upregulation of mineralocorticoids [8, 18]. Moreover, many steroids have intrinsic mineralocorticoid activities [19], which may excessively stimulate mineralocorticoid receptors (MR). Previous studies have identified three main processes linking MR overactivation with cardiovascular diseases, namely oxidative stress, inflammation and fibrosis, which can cause HF, electrophysiological disturbances and vascular diseases such as chronic hypertension [20]. Since in vitro evidence suggests that MR expression in the heart increases with age [21], abiraterone use in elderly may lead to increased MR activation and significantly increase the risk of 4P-MACE. By contrast, enzalutamide works by targeting the androgen receptor signaling pathway [22]. As it does not inhibit androgen synthesis, there is no mineralocorticoid excess and its disposition to adverse cardiac events.

It is worthy to note that enzalutamide, when compared with abiraterone, appears to be associated with a significantly reduced cardiovascular risk in non-diabetic but not in diabetic patients. Firstly, diabetic patients may develop substantial macro- and micro- vascular complications via mechanisms such as the formation of advanced glycation end-products, and activation of protein kinase C [23]. These can increase the cardiovascular risk in many ways, including the release of pro-inflammatory molecules, increased vascular permeability, occlusion of vasculature and excessive apoptosis [24, 25]. As a result, the ‘testosterone bounce’ brought by enzalutamide may not be effective in reversing the substantial vascular complications of diabetes. Secondly, an in vitro study has suggested that diabetes mellitus downregulates BKCa channel beta1-subunits, thus leading to reduced activity of the BKCa channels [26]. As such, the vasodilatory effect of enzalutamide-induced ‘testosterone bounce’ may be diminished in patients with diabetes, thus unable to prevent 4P-MACE [27].

### Prior studies and future directions

Randomized controlled trials (RCTs) have explored the risks for adverse cardiac events for enzalutamide or abiraterone individually or when compared to placebo [8, 28]. Nonetheless, we further compared the two drugs directly and studied adverse cardiac events individually. Subsequently, a meta-analysis of randomized controlled trials used surface under the cumulative ranking curve (SUCRA) to show that abiraterone may be associated with higher risks for adverse cardiac events than enzalutamide, while enzalutamide may be associated with higher risks for hypertension [29]. Nonetheless, SUCRA values are largely dependent on estimated effect sizes, which are variable [30]. On the other hand, our study directly compared the risks for 4P-MACE between enzalutamide and abiraterone in clinical practise using a representative territory-wide cohort. In a recent observational study, Kulkarni et al. reported an increased risk for stroke and MI associated with abiraterone when compared with enzalutamide [31]. By additionally including HF as an outcome and adjusting for residual confounding using PS weighting, our study more thoroughly demonstrates that enzalutamide has a better cardiac risk profile. In addition, the intention-to-treat analysis used in Kulkarni et al. may not be suitable in observational studies [31, 32], as it could underestimate drug-associated cardiotoxicities especially when related to the reason for drug switching.

In addition, the cardiotoxicities of enzalutamide and abiraterone combination merits further exploration. While initial reports suggested modest improvements in progression-free survival for the combined use of these two drugs in metastatic CRPC [33, 34], the indicators for adverse cardiac events were not demonstrated in detail, probably due to the small number of patients receiving both drugs that limits any useful statistical analyses to be performed. Currently, relevant ongoing trials are being conducted (NCT01650194 and NCT01949337). Nonetheless, this gap in evidence remains open and awaits further investigations. Also, patients with non-metastatic cancer experiencing biochemical recurrence after local therapies are often under-represented in existing literature. While enzalutamide and abiraterone have increasingly shown survival benefits in patients with non-metastatic PCa [5], little is known regarding their cardiovascular risks in this setting which could be a barrier to their use in non-metastatic PCa.

Our findings should further prompt clinicians to consider the relative risks of MACE when prescribing enzalutamide or abiraterone. The cardiovascular risk profiles of patients with PCa should also be taken into account in choosing these medications. Although RCTs using these drugs to evaluate cardiac outcomes in their prospective studies are needed to confirm our findings, prospective data may aid in shared decision-making.

### Strengths and limitations

This study used a representative population-based database with a long follow-up duration. Our results are, therefore, likely to be widely generalizable and reflect real-world practice. Sensitivity analyses using different approaches were performed showing consistent results, indicating robustness. However, several limitations should be noted. Firstly, as an observational study, residual confounding cannot be excluded. Secondly, since all diagnoses were identified using ICD-9 codes as recorded by CDARS, the data could not be adjudicated individually. Nonetheless, all diagnostic codes were input by treating clinicians independent of the authors, and previous studies of CDARS have shown good coding accuracy. Thirdly, whilst concurrent prescriptions within the same period can be obtained, it was not possible to ascertain the reason for such prescriptions. For example, steroids may be prescribed for reasons other than prostate cancer even if prescribed by an urologist. Given that co-prescription of abiraterone and steroid is a common clinical practise worldwide, we restricted the abiraterone arm to those with steroid use on the day of abiraterone initiation. Fourthly, cancer staging is lacking due to a lack of coding of this information in CDARS. Nonetheless, enzalutamide and abiraterone were introduced at similar times in our locality. According to local treatment guidelines, both drugs are prescribed to patients presented with castration-resistant prostate cancer (CRPC), and to patients with metastatic hormone-sensitive prostate cancer (mHSPC) since a few years ago. Therefore, we would not expect a significant difference in terms of usage of the two drugs in mHSPC or mCRPC stages.

## Conclusion

Enzalutamide use is associated with significantly lower risks for 4P-MACE, MI, and all-cause mortality compared to abiraterone use in patients with PCa.

## Supplementary information

Suppl

## Data Availability

All data underlying this study are available on reasonable request to the corresponding authors.

## References

[1] Wang L, Lu B, He M, Wang Y, Wang Z, Du L. Prostate cancer incidence and mortality: global status and temporal trends in 89 countries from 2000 to 2019. Front Public Health. 2022;10:811044. 35252092 10.3389/fpubh.2022.811044 PMC8888523

[2] Chandrasekar T, Yang JC, Gao AC, Evans CP. Mechanisms of resistance in castration-resistant prostate cancer (CRPC). Transl Androl Urol. 2015;4:365–80. 26814148 10.3978/j.issn.2223-4683.2015.05.02 PMC4708226

[3] Fizazi K, Tran N, Fein L, Matsubara N, Rodriguez-Antolin A, Alekseev BY, et al. Abiraterone plus prednisone in metastatic, castration-sensitive prostate cancer. N Engl J Med. 2017;377:352–60. 28578607 10.1056/NEJMoa1704174

[4] Davis ID, Martin AJ, Stockler MR, Begbie S, Chi KN, Chowdhury S, et al. Enzalutamide with standard first-line therapy in metastatic prostate cancer. N Engl J Med. 2019;381:121–31. 31157964 10.1056/NEJMoa1903835

[5] Attard G, Murphy L, Clarke NW, Cross W, Jones RJ, Parker CC, et al. Abiraterone acetate and prednisolone with or without enzalutamide for high-risk non-metastatic prostate cancer: a meta-analysis of primary results from two randomised controlled phase 3 trials of the STAMPEDE platform protocol. Lancet. 2022;399:447–60. 34953525 10.1016/S0140-6736(21)02437-5 PMC8811484

[6] Scher HI, Fizazi K, Saad F, Taplin ME, Sternberg CN, Miller K, et al. Increased survival with enzalutamide in prostate cancer after chemotherapy. N Engl J Med. 2012;367:1187–97. 22894553 10.1056/NEJMoa1207506

[7] Tan PS, Haaland B, Montero AJ, Kyriakopoulos CE, Lopes G. Hormonal therapeutics enzalutamide and abiraterone acetate in the treatment of metastatic castration-resistant prostate cancer (mCRPC) post-docetaxel-an indirect comparison. Clin Med Insights Oncol. 2014;8:29–36. 24678245 10.4137/CMO.S13671 PMC3964205

[8] Iacovelli R, Ciccarese C, Bria E, Romano M, Fantinel E, Bimbatti D, et al. The cardiovascular toxicity of abiraterone and enzalutamide in prostate cancer. Clin Genitourin Cancer. 2018;16:e645–53. 29339044 10.1016/j.clgc.2017.12.007

[9] Lee YHA, Zhou J, Hui JMH, Liu X, Lee TTL, Hui K, et al. Risk of new-onset prostate cancer for metformin versus sulfonylurea use in type 2 diabetes mellitus: a propensity score–matched study. J Natl Compr Cancer Netw. 2022;20:674–82.e15. 10.6004/jnccn.2022.7010 35714677

[10] Chan JSK, Satti DI, Lee YHA, Hui JMH, Lee TTL, Chou OHI, et al. High visit-to-visit cholesterol variability predicts heart failure and adverse cardiovascular events: a population-based cohort study. Eur J Prev Cardiol. 2022;29:e323–5. 35653641 10.1093/eurjpc/zwac097

[11] Chan JSK, Lee YHA, Liu K, Hui JMH, Dee EC, Ng K, et al. Long-term cardiovascular burden in prostate cancer patients receiving androgen deprivation therapy. Eur J Clin Invest. 2023;53:e13932. 36468787 10.1111/eci.13932

[12] Lee YHA, Hui JMH, Chan JSK, Liu K, Dee EC, Ng K, et al. Metformin use and mortality in Asian, diabetic patients with prostate cancer on androgen deprivation therapy: a population-based study. Prostate. 2023;83:119–27. 36178848 10.1002/pros.24443 PMC9742285

[13] Bosco E, Hsueh L, McConeghy KW, Gravenstein S, Saade E. Major adverse cardiovascular event definitions used in observational analysis of administrative databases: a systematic review. BMC Med Res Methodol. 2021;21:241. 34742250 10.1186/s12874-021-01440-5 PMC8571870

[14] McCaffrey DF, Ridgeway G, Morral AR. Propensity score estimation with boosted regression for evaluating causal effects in observational studies. Psychol Methods. 2004;9:403–25. 15598095 10.1037/1082-989X.9.4.403

[15] Sturmer T, Wyss R, Glynn RJ, Brookhart MA. Propensity scores for confounder adjustment when assessing the effects of medical interventions using nonexperimental study designs. J Intern Med. 2014;275:570–80. 24520806 10.1111/joim.12197 PMC4037382

[16] Lester JF, Mason MD. Cardiovascular effects of hormone therapy for prostate cancer. Drug Health Patient Saf. 2015;7:129–38. 10.2147/DHPS.S50549 PMC4516188 26229507

[17] Nordrehaug JE, Johannessen KA, von der Lippe G. Serum potassium concentration as a risk factor of ventricular arrhythmias early in acute myocardial infarction. Circulation. 1985;71:645–9. 3971535 10.1161/01.cir.71.4.645

[18] Attard G, Merseburger AS, Arlt W, Sternberg CN, Feyerabend S, Berruti A, et al. Assessment of the safety of glucocorticoid regimens in combination with abiraterone acetate for metastatic castration-resistant prostate cancer: a randomized, open-label phase 2 study. JAMA Oncol. 2019;5:1159–67. 31246234 10.1001/jamaoncol.2019.1011 PMC6604092

[19] Bretagne M, Lebrun-Vignes B, Pariente A, Shaffer CM, Malouf GG, Dureau P, et al. Heart failure and atrial tachyarrhythmia on abiraterone: a pharmacovigilance study. Arch Cardiovasc Dis. 2020;113:9–21. 31685432 10.1016/j.acvd.2019.09.006

[20] Buonafine M, Bonnard B, Jaisser F. Mineralocorticoid receptor and cardiovascular disease. Am J Hypertens. 2018;31:1165–74. 30192914 10.1093/ajh/hpy120

[21] Hu D, Dong R, Zhang Y, Yang Y, Chen Z, Tang Y, et al. Agerelated changes in mineralocorticoid receptors in rat hearts. Mol Med Rep. 2020;22:1859–67. 32582979 10.3892/mmr.2020.11260 PMC7411371

[22] Urata T. [Life science: “medicine and man”. Genetic factors and the clinical course in a disease]. Kango Kyoshitsu. 1974;18:34–7. 4492664

[23] Gleissner CA, Galkina E, Nadler JL, Ley K. Mechanisms by which diabetes increases cardiovascular disease. Drug Discov Today Dis Mech. 2007;4:131–40. 18695749 10.1016/j.ddmec.2007.12.005 PMC2504760

[24] Singh VP, Bali A, Singh N, Jaggi AS. Advanced glycation end products and diabetic complications. Korean J Physiol Pharm. 2014;18:1–14. 10.4196/kjpp.2014.18.1.1 PMC3951818 24634591

[25] Das Evcimen N, King GL. The role of protein kinase C activation and the vascular complications of diabetes. Pharm Res. 2007;55:498–510. 10.1016/j.phrs.2007.04.016 17574431

[26] Wang Y, Zhang HT, Su XL, Deng XL, Yuan BX, Zhang W, et al. Experimental diabetes mellitus down-regulates large-conductance Ca2+-activated K+ channels in cerebral artery smooth muscle and alters functional conductance. Curr Neurovasc Res. 2010;7:75–84. 20334613 10.2174/156720210791184925

[27] Deenadayalu V, Puttabyatappa Y, Liu AT, Stallone JN, White RE. Testosterone-induced relaxation of coronary arteries: activation of BKCa channels via the cGMP-dependent protein kinase. Am J Physiol Heart Circ Physiol. 2012;302:H115–23. 22081702 10.1152/ajpheart.00046.2011 PMC3334243

[28] Moreira RB, Debiasi M, Francini E, Nuzzo PV, Velasco G, Maluf FC, et al. Differential side effects profile in patients with mCRPC treated with abiraterone or enzalutamide: a meta-analysis of randomized controlled trials. Oncotarget. 2017;8:84572–8. 29137449 10.18632/oncotarget.20028 PMC5663621

[29] Lee HY, Chen HL, Teoh JY, Chen TC, Hao SY, Tsai HY, et al. Abiraterone and enzalutamide had different adverse effects on the cardiovascular system: a systematic review with pairwise and network meta-analyses. Prostate Cancer Prostatic Dis. 2021;24:244–52. 32860011 10.1038/s41391-020-00275-3

[30] Salanti G, Nikolakopoulou A, Efthimiou O, Mavridis D, Egger M, White IR. Introducing the treatment hierarchy question in network meta-analysis. Am J Epidemiol. 2022;191:930–8. 35146500 10.1093/aje/kwab278 PMC9071581

[31] Kulkarni AA, Rubin N, Tholkes A, Shah S, Ryan CJ, Lutsey PL, et al. Risk for stroke and myocardial infarction with abiraterone versus enzalutamide in metastatic prostate cancer patients. ESMO Open. 2021;6:100261. 34509804 10.1016/j.esmoop.2021.100261 PMC8437777

[32] Pazzagli L, Linder M, Zhang M, Vago E, Stang P, Myers D, et al. Methods for time-varying exposure related problems in pharmacoepidemiology: an overview. Pharmacoepidemiol Drug Saf. 2018;27:148–60. 29285840 10.1002/pds.4372 PMC5814826

[33] Morris MJ, Heller G, Bryce AH, Armstrong AJ, Beltran H, Hahn OM, et al. Alliance A031201: a phase III trial of enzalutamide (ENZ) versus enzalutamide, abiraterone, and prednisone (ENZ/AAP) for metastatic castration resistant prostate cancer (mCRPC). J Clin Oncol. 2019;37:5008. 10.1200/JCO.22.02394 PMC10414728 36996380

[34] Saad F, Efstathiou E, Attard G, Flaig TW, Franke F, Goodman OB Jr, et al. Apalutamide plus abiraterone acetate and prednisone versus placebo plus abiraterone and prednisone in metastatic, castration-resistant prostate cancer (ACIS): a randomised, placebo-controlled, double-blind, multinational, phase 3 study. Lancet Oncol. 2021;22:1541–59. 34600602 10.1016/S1470-2045(21)00402-2 PMC9377412

