# Supplementary material for: Major adverse cardiovascular events of enzalutamide versus abiraterone in prostate cancer: a retrospective cohort study
Source: Prostate Cancer Prostatic Dis. 2023 Dec 5;27(4):776–82. doi: 10.1038/s41391-023-00757-0 (PMC11543592; doi:10.1038/s41391-023-00757-0)
Supplement: Supplementary file 1 — Suppl [file 41391_2023_757_MOESM1_ESM.pdf]

Supplementary Table 1. ICD-9 codes used to identify outcomes and co-morbidities. All hereby listed codes include the corresponding sub-codes.

Prostate cancer	185
Diabetes mellitus	250
Hypertension	401 402 403 404 405 437.2
Ischaemic heart disease	410 411 412 413 414
Myocardial infarction	410
Heart failure	428
Stroke or transient ischaemic attack	430 431 432 433 434 435
Chronic kidney disease	582 585 586
Hyperlipidaemia	272.0 272.1 272.2 272.3 272.4
Other malignancies	140 141 142 143 144 145 146 147 148 149<br>150 151 152 153 154 155 156 157 158<br>159 160 161 162 163 164 165 170 171<br>172 173 174 175 179 179 180 181 182<br>183 184 185 186 187 188 189 190 191

	192 193 194 195 196 197 198 199 200<br>201 202 203 204 205 206 207 208<br>209.0 209.1 209.2 209.3

ICD-9, *International Classification of Diseases, Ninth Revision*.

Supplementary Table 2. Summary of Main Analyses, with wHR referenced against Abiraterone Use.

	**wHR (95% CI)**	**P-value**
4P-MACE	0.71 (0.59 – 0.86)	<0.001
MI	0.57 (0.33 – 0.97)	0.040
Stroke	1.18 (0.58 – 2.39)	0.648
HF	0.43 (0.16 – 1.15)	0.091
All-cause mortality	0.71 (0.59 – 0.85)	<0.001

4P-MACE, 4-point major adverse cardiovascular events. CI, confidence interval. HF, heart failure. MI, myocardial infarction. wHR, weighted hazard ratio.

Supplementary Table 3. Multivariable Analysis for 4P-MACE, with aHR referenced against Abiraterone Use.

	**aHR (95% CI)**	**P-value**
4P-MACE	0.71 (0.60 – 0.84)	<0.001

4P-MACE, 4-point major adverse cardiovascular events. aHR, adjusted hazard ratio.CI, confidence interval.
